# Latissimus dorsi flap – the main force in breast reconstruction for breast tumor in Chinese population

**DOI:** 10.3389/fonc.2023.1159073

**Published:** 2023-07-20

**Authors:** Shuyue Zheng, Shuang Hao, Jiajian Chen, Yingying Zhang, Benlong Yang, Xiaoyan Huang, Guangyu Liu, Zhimin Shao, Jiong Wu

**Affiliations:** ^1^Department of Breast Surgery, Fudan University Shanghai Cancer Center, Shanghai, China; ^2^Department of General Surgery, Zhongshan Hospital, Fudan University, Shanghai, China; ^3^Department of Oncology, Shanghai Medical College, Fudan University, Shanghai, China

**Keywords:** breast tumor, latissimus dorsi flaps, implants, breast reconstruction, complications

## Abstract

**Background:**

The latissimus dorsi flap (LDF) is the most commonly used autologous flap for breast reconstruction (BR) in China. We conducted this study to explore the current status of BR using LDF with/without implants.

**Methods:**

This study was a single-center retrospective study that included breast tumor patients who underwent LDF breast reconstruction at Fudan University Shanghai Cancer Center (FUSCC) between 2000 and 2021.

**Results:**

We analyzed 4918 patients who underwent postmastectomy BR, including 1730 patients (35.2%) with autologous flaps. LDF was used for BR in 1093 (22.2%) patients, and an abdominal flap was used in 637 (13.0%) patients. The proportion of LDFs used in autologous BR patients decreased each year and dropped to approximately 65.0% after 2013 due to the increased use of abdominal flaps. Among these patients, 609 underwent extended LDF (ELDF) BR, 455 underwent LDF BR with implants, and 30 received a LDF as a salvage flap due to previous flap or implant failure. Patients who underwent ELDF reconstruction were older and had a higher BMI than those who received a LDF with implants. There was no significant difference in the mean postoperative hospital stay, neoadjuvant chemotherapy rates, or adjuvant radiotherapy rates between the two groups. Major complications requiring surgical intervention occurred in 25 patients (2.29%). There was no significant difference in the incidence of major complications between the two groups (*P*=0.542).

**Conclusions:**

LDF breast reconstruction is a well-developed and safe procedure. The duration of postoperative hospitalization nor the incidence of major complications was affected by implant use.

## Introduction

Owing to increasing public awareness and the development of screening programs, more patients with breast cancer can be diagnosed at much earlier stages, and the survival rate of these patients has greatly increased (1, 2). Patients have become increasingly concerned about changes in body shape and complain about diminished femininity and self-confidence after mastectomy. Thus, breast reconstruction has become an important part of breast cancer management and can improve patient satisfaction without affecting multidisciplinary adjuvant treatment (3).

Implant-only breast reconstruction remains the most performed type of immediate breast reconstruction, but autologous techniques involving donor sites account for approximately 20% (4). The common donor sites for autologous breast reconstruction are the latissimus dorsi flap (LDF) and the abdominal flap. Kamali et al. conducted a retrospective study using the Nationwide Inpatient Sample database (2008 to 2012), which showed a trend of a significant increase in the use of the LDF both nationally (P < 0.001) and regionally (P < 0.001) (4). Asian women have relatively smaller breasts than European and American women, so the latissimus dorsi flap (LDF) is the most used autologous flap for breast reconstruction in Chinese patients with breast tumors. In 2017, a nationwide cross-sectional survey of 110 hospitals conducted in China showed that among patients with autologous flap reconstruction, 69.8% (1562 cases in 2238 cases) underwent LDF reconstruction, including 625 extended latissimus dorsi flaps (ELDFs) and 927 LDFs with implants (5). Compared to abdominal flaps, latissimus dorsi flaps are beneficial in that they have reliable vessel distribution, favorable proximity to defects and simple dissection, so effective application requires a relatively shorter learning curve for surgeons and a shorter operating time.

Although several researchers have reported successful clinical outcomes after autologous flap breast reconstruction to date, there is a lack of large-scale studies in which researchers focus on the development of LDF breast reconstruction over time in China (6). We conducted this study to explore the status of breast reconstruction using LDF with or without implants (IMP) and the potential factors influencing the choice of reconstruction procedure.

## Methods

Patients with breast tumors who underwent breast reconstruction after mastectomy at Fudan University Shanghai Cancer Center (FUSCC) between 2000 and 2021 were included in the retrospective study. Patients who underwent breast reconstruction using LDF with/without implants were analyzed. This study was approved by the Ethics Committee of FUSCC (Shanghai, China; ID: 1612167-18) and conducted in accordance with the Declaration of Helsinki. Informed consent was not needed for the retrospective study.

Female patients aged 18 to 85 years with breast tumors who underwent postmastectomy breast reconstruction were analyzed in this study. Patients who underwent prophylactic mastectomy, breast-conserving surgery, partial reconstruction, poor cardiopulmonary function or with contraindications to surgery were excluded. We obtained data on basic clinicopathological information, the timing and type of reconstruction, the duration of postoperative hospitalization and the incidence of major complications requiring surgical intervention. The clinical and pathological stages of patients included in the analysis were classified according to the AJCC version 8. The pathological diagnoses were confirmed independently by two expert pathologists. Only patients with invasive carcinoma were classified by pathological tumor stage (pT), pathological nodal stage (pN), estrogen receptor (ER) status, progesterone receptor (PR) status, pathologic HER2 status and Ki67 status. Pathologic HER2 status was defined according to the ASCO/CAP 2007 guidelines (7). For ER and PR, ≥1% of cells with strongly stained tumor nuclei were considered positive, and <1% were considered negative (8). For a more accurate analysis of postoperative complications, only those requiring reoperation were included.

For patients planned for immediate breast reconstruction, standard skin sparing mastectomy (SSM) or nipple sparing mastectomy (NSM) was performed. Autologous breast reconstruction included latissimus dorsi flaps with or without implants and abdominal flap breast reconstruction, including pedicle transverse rectus abdominis myocutaneous (pTRAM) flaps, and free tissue transfers using abdominal flaps, including free TRAM, muscle-sparing free TRAM, and deep inferior epigastric perforator flap (DIEP). Implant-only based breast reconstruction included tissue-expander prosthetic placement and prosthetic placement alone.

Continuous variables are expressed as the mean values or median values, and categorical variables are expressed as frequencies. The consecutive variables were analyzed by t test. Categorical variables were analyzed by using Chi-square and Kruskal−Wallis tests. Multivariate logistical regression analysis was performed to determine factors associated with the selection of LDF breast reconstruction with or without implants. Statistical analyses were performed using SPSS version 26. All P values reported were two-sided and were calculated at a significance level of 0.05.

## Results

### Trends in breast reconstruction technology from 2000 to 2021

**Figure 1:**
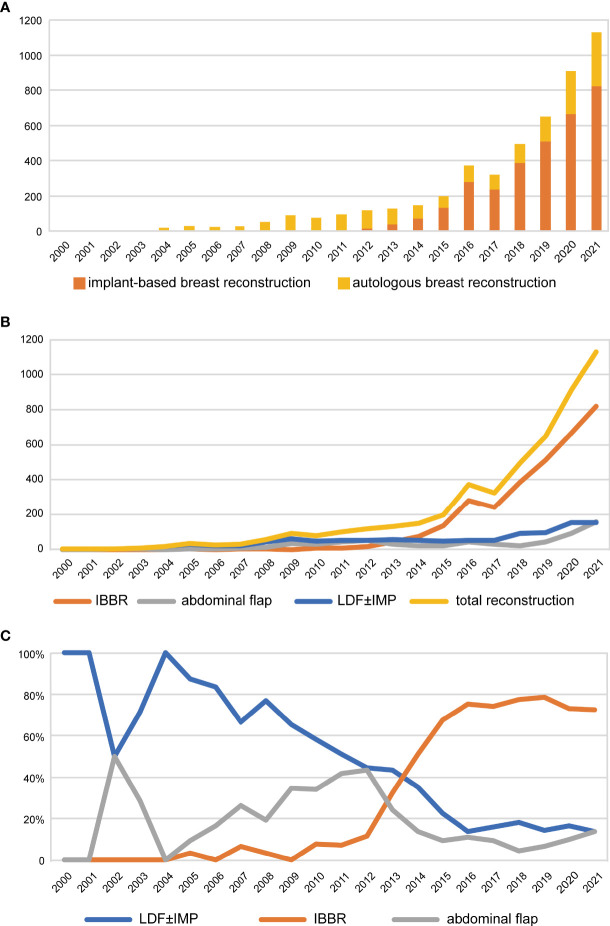
Trends of breast reconstruction from 2000 to 2021. **(A)** Trends of the proportion of IBBR and autologous breast reconstruction over years. **(B)** Trends of different types of breast reconstruction over years. **(C)** Trends of the proportion of different types in all breast reconstruction over years. LDF, latissimus dorsi flap; IMP, implants; IBBR, implant-based breast reconstruction.

**Figure 2:**
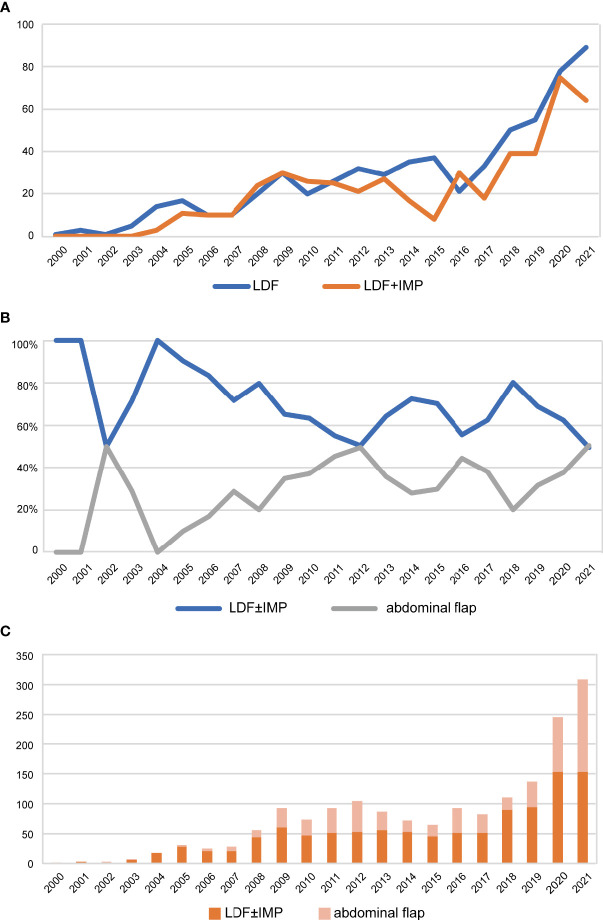
Trends of autologous breast reconstruction from 2000 to 2021. **(A)** Trends of LDF and LDF+implant breast reconstruction over years. **(B)** Trends of the proportion of LDF ± implant and abdominal flap in autologous breast reconstruction over years. **(C)** Histogram of the proportion of LDF ± implant and abdominal flap to autologous reconstruction over years. LDF, latissimus dorsi flap; IMP, implants.

From 2000 to 2021, a total of 4918 patients with breast tumors underwent postmastectomy breast reconstruction at FUSCC. The proportion of reconstruction in total breast tumor surgery has increased year by year, reaching 13.7% in the past 2 years. Advances in breast reconstructive techniques have broadened the postmastectomy reconstruction choices for female patients. Significant progress has been made in both implant- and autologous-based breast reconstruction in the past 22 years (**Figures 1A, B**). During the past 22 years, the proportion of autologous-based breast reconstructions performed in patients who underwent postmastectomy breast reconstruction gradually decreased to a relatively stable proportion of approximately 24.7% after 2016 due to IBBR being increasingly performed, and the proportion of LDF breast reconstructions decreased to approximately 15.4% (**Figure 1C**). The proportion of LDFs used in autologous breast reconstruction patients also decreased each year and dropped to a relatively stable proportion of approximately 65.0% after 2013 due to the increased use of abdominal flaps (**Figures 2A, B**). Of all reconstruction cases, 35.2% were autologous reconstruction, and LDF was the most popular option for use in autologous reconstruction (**Figure 2C**).

### Current status of LDF breast reconstruction

In a total of 1093 (63.2% of autologous reconstruction patients) patients, a LDF was used for breast reconstruction, and in 637 (36.8%) patients, abdominal flaps were used for autologous breast reconstructions. Among patients who underwent LDF breast reconstruction, most (1043/1093, 95.42%) underwent immediate breast reconstruction, only 29 (2.65%) underwent delayed reconstruction and 21 (1.92%) underwent immediate-delayed reconstruction. In patients who underwent immediate-delayed reconstruction, an expander was implanted immediately after mastectomy. After inflating the expander with saline over a period of time or adjuvant radiotherapy, the expander was then replaced by a LDF with or without a permanent implant. A total of 615 patients underwent extended LDF breast reconstruction, and 478 (43.6%) patients underwent LDF combined with implant breast reconstruction, including 34 patients who underwent two-stage expander-to-implant LDF flap breast reconstruction. Thirty patients received a LDF as a salvage flap in cases of previous flap or implant failure and chest wall defects. Among these patients, 27 received a LDF due to unsatisfactory outcomes of expander reconstruction or failed implant reconstruction, including 11 patients who underwent ELDF reconstruction and 16 patients who underwent LDF with implant reconstruction. One patient received a LDF to repair a poorly healed breast incision, and two patients underwent LDF reconstruction due to failed abdominal flap reconstruction, including one TRAM and one DIEP.

The mean age of the patients at the time of breast reconstruction surgery was 38.14 years (range 19 to 77). The mean BMI of the LDF reconstruction patients was 22.30 kg/cm2. The mean duration of postoperative hospitalization was 9.41 days. In terms of pathological type, 925 (84.63%) patients had invasive carcinoma, 135 (12.35%) had carcinoma in situ, 21 (1.92%) patients had phyllodes tumors and 11 (1.90%) patients had other malignancies. There were 264 (24.15%) patients treated with neoadjuvant chemotherapy and 356 (32.57%) treated with adjuvant radiotherapy.

**Figure 3:**
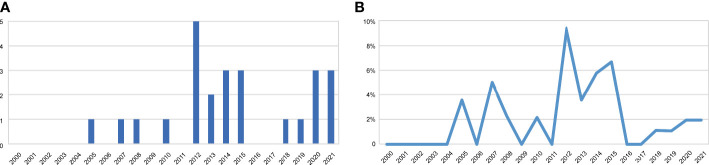
The trend of major complications requiring surgical intervention over the years. **(A)** The incidence of major complications. **(B)** The cases of major complications.

Major complications requiring surgical intervention occurred in 25 patients (2.29%). The incidence of major complications over the years is shown in **Figure 3**, and the list is shown in **Table 1**. Fourteen patients underwent surgical debridement or scar revision due to wound infection or poor healing, 8 patients underwent implant or flap removal due to serious infection or vascular crisis, and one patient underwent breast reconstruction revision operations concurrently with nipple reconstruction. The reconstructed breast and implant were removed in 1 patient due to the recurrence of breast cancer. One patient requested replacement of a smaller prosthesis 10 years after reconstruction because she perceived that the reconstructed breast was too large.

**Table 1:** Cases with complications requiring surgical intervention.

Type of reconstruction		Complications	Solution	Time after surgery
LDF+implant	1	Postoperative hemorrhage	Debridement and hemostasis	1 day
	2	Reconstructed latissimus dorsi flap venous crisis	Debridement, hemostasis and remove the implant	5 days
	3	Implant infection	Remove implant	8 days
	4	Prosthesis exposure	Remove the prosthesis	4 months
	5	Insufficient expansion volume	Remove the expander	1 year
	6	Breast cancer recurrence	Remove the reconstructed breast and prosthesis	2 years
	7	Reconstructed breast pain	Remove implant	6 years
	8	Perceived prosthesis too large	Replace a smaller prosthesis	10 years
	9	Implant infection	Remove implant	2 years
LDF	1	Postoperative hemorrhage	Debridement and hemostasis	2 days
	2	Back incision dehiscence	Debridement and suturing	1month
	3	Back incision dehiscence	Debridement and suturing	7 months
	4	Back incision poor healing	Suturing	12 days
	5	Poor incision healing	Debridement and suturing	4 months
	6	Incision not healing	Debridement and suturing	2 months
	7	Reconstructed breast fat liquefaction	Debridement	2 months
	8	Seroma in back	Debridement	6 months
	9	Breast incision scarring	Scar excision	4 months
	10	Back incision scarring	Scar excision	6 months
	11	LDF necrosis	Remove the reconstructed breast	2 months
	12	Vascular crisis	Remove the graft flap	1 day
	13	Back incision dehiscence	Debridement and suturing	7 months
	14	Reconstructed breast deformity	Revision	1 year
	15	Postoperative hemorrhage	Debridement and hemostasis	1 day
	16	Sinus tract formation of back incision	Flap repair	4 months

### Potential influencing factors of LDF combined with or without implants

The clinicopathological information of patients who underwent postmastectomy LDF breast reconstruction with or without implants is summarized in **Table 2**. Compared with the LDF combined with implants group, the patients in the LDF without implants breast reconstruction group were older (39.46 vs. 36.44, *P <*0.0001) and had a higher mean BMI (22.16 ± 2.91 kg/cm^2^ vs. 21.60 ± 2.65 kg/cm^2^, *P*=0.001). In terms of reconstruction timing, 97.4% of ELDF breast reconstructions were immediate, only 2.3% of ELDF reconstructions were delayed, and 0.3% were immediate-delayed. For patients treated with a LDF combined with implants, 19 (4.0%) were immediate-delayed, and 15 (3.1%) were delayed. The patients with phyllodes tumors and other malignant tumors tended to undergo extended latissimus dorsi flap reconstruction (*P*=0.021). There were no significant differences in the duration of postoperative hospitalization (*P*=0.540), the rate of neoadjuvant chemotherapy (*P*=0.569), or the rate of adjuvant radiotherapy (*P*=0.852) between patients treated with ELDF breast reconstruction and those treated with a LDF combined with implants. There was also no significant difference in the incidence of major complications requiring surgical intervention between the patients who underwent extended LDF and those who underwent LDF combined with implant reconstruction, *P*=0.542.

**Table 2:** The clinicopathological information of patients underwent LDF with or without implants breast reconstruction.

Characteristics	ELDF	LDF+implant	*P*-valu*e*
	N(%)	N(%)	
Postoperative hospital stay, Mean ± SD	9.50 ± 5.90	9.29 ± 5.37	0.540
Age, Mean ± SD	39.46 ± 8.56	36.44 ± 7.52	<0.0001
Age			0.002
<35	201(32.7%)	200(41.8%)	
35-64	406(66.0%)	277(57.9%)	
>64	8(1.3%)	1(0.2%)	
BMI Mean ± SD	22.16 ± 2.91	21.60 ± 2.65	0.001
BMI			
<18.5	43(7.0%)	46(9.6%)	0.213
18.5-28	546(88.8%)	418(87.4%)	
>28	22(3.6%)	10(2.1%)	
Unknown	4(0.7%)	4(0.8%)	
Neoadjuvant therapy			0.569
No	462(75.1%)	367(76.8%)	
Yes	153(24.9%)	111(23.2%)	
Pathological types			0.021
Invasive carcinoma	515(83.7%)	410(85.8%)	
Carcinoma in situ	73(11.9%)	62(13.0%)	
Phyllodes tumors	19(3.1%)	3(0.6%)	
Other tumors	8(1.3%)	3(0.6%)	
Timing of reconstruction			<0.0001
Immediately	599(97.4%)	444(92.9%)	
Delay	142(2.3%)	15(3.1%)	
Immediate to delay	2(0.3%)	19(4.0%)	
Radiotherapy			0.852
Yes	199(32.4%)	157(32.8%)	
No	341(55.4%)	268(56.1%)	
Unknown	75(12.2%)	53(11.1%)	
Complications			0.542
No	599(97.4%)	469(98.1%)	
Yes	16(2.6%)	9(1.9%)	

**Figure 4:**
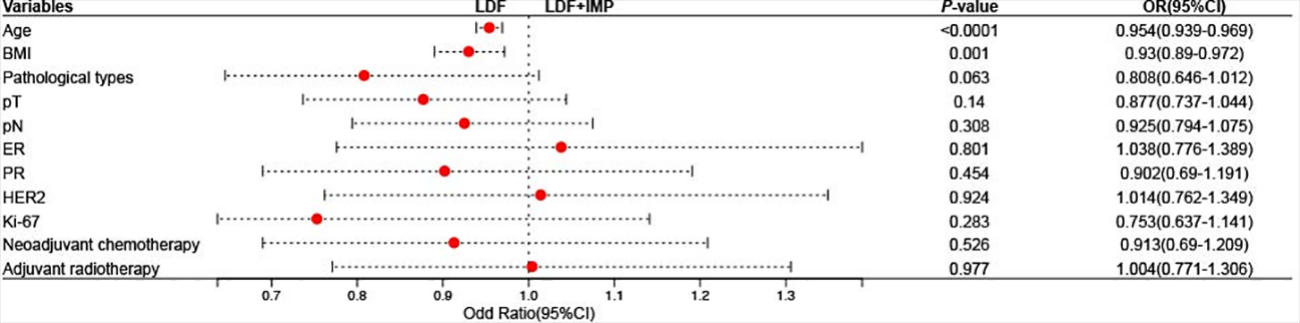
Forest plots for the potential influencing factors of the type of reconstruction.

Among the patients diagnosed with invasive carcinoma, there was no significant difference in pT, pN, ER, PR, HER2, Ki67, or the rate of neoadjuvant chemotherapy and adjuvant radiotherapy between the patients who underwent extended LDF and those who underwent LDF combined with implant breast reconstruction (**Table 3**). To further explore the potential factors influencing the choice of reconstruction procedure, we performed univariate logistical regression analysis and constructed forest plots. The results showed that age and BMI were important factors influencing whether LDF combined with implant breast reconstruction was chosen (**Figure 4**). Multivariate logistic regression analysis was performed using the factors that showed significance in the univariate logistic regression analysis, and the results suggested that age (OR (95% CI): 0.957 (0.942-0.973), *P*<0.0001) and BMI (OR (95% CI): 0.948 (0.907-0.991), *P*=0.019) were independent factors influencing the choice of LDF or LDF combined with implant breast reconstruction.

**Table 3:** The clinicopathological information of patients diagnosed with invasive carcinoma.

Characteristics	ELDF	LDF+implant	*P*-valu*e*
	N(%)	N(%)	
Postoperative hospital stay Mean ± SD	9.22 ± 5.70	9.09 ± 5.35	0.722
Age, Mean ± SD	39.40 ± 8.50	36.32 ± 7.66	<0.0001
Age			0.002
<35	173(33.6%)	181(44.1%)	
35-64	336(65.2%)	228(55.6%)	
>64	6(1.2%)	1(0.2%)	
BMI Mean ± SD	22.31 ± 2.99	21.72 ± 2.61	0.002
BMI			
<18.5	37(7.2%)	37(9.0%)	0.156
18.5-28	453(88.0%)	361(88.0%)	
>28	22(4.3%)	8(2.0%)	
Unknown	3(0.6%)	4(1.0%)	
Neoadjuvant therapy			0.461
No	366(71.1%)	301(73.4%)	
Yes	149(28.9%)	109(26.6%)	
pT			0.239
0	44(8.5%)	32(7.8%)	
1	216(41.9%)	203(49.5%)	
2	179(34.8%)	121(29.5%)	
3	46(8.9%)	31(7.6%)	
Unknown	30(5.8%)	23(5.6%)	
pN			0.485
0	278(54.0%)	232(56.6%)	
1	142(27.6%)	105(23.6%)	
2	60(11.7%)	54(13.2%)	
3	29(5.6%)	14(3.4%)	
Unknown	6(1.2%)	5(1.2%)	
ER			0.857
–	144(28.0%)	111(27.1%)	
+	352(68.3%)	286(69.8%)	
Unknown	19(3.7%)	13(3.2%)	
PR			0.750
–	190(36.9%)	161(39.3%)	
+	307(59.6%)	236(57.6%)	
Unknown	18(3.5%)	13(3.2%)	
Her2			0.510
–	323(62.8%)	250(61.0%)	
+	158(30.7%)	125(30.5%)	
Unknown	33(6.4%)	35(8.5%)	
Ki-67			0.386
<20%	158(30.7%)	136(33.2%)	
≥20%	272(52.8%)	198(48.3%)	
Unknown	85(16.5%)	76(18.5%)	
Radiotherapy			0.924
Yes	275(53.4%)	217(52.9%)	
No	184(35.7%)	150(36.6%)	
Unknown	56(10.9%)	43(10.5%)	
Major complications			0.304
Yes	16(3.1%)	8(2.0%)	
No	499(96.9%)	402(98.0%)	

## Discussion

Breast reconstruction retains the shape of the breast and significantly improves the quality of life and increases the confidence of patients after mastectomy (9, 10) In this study, we analyzed the trends of breast reconstruction performed in China from 2000 to 2021 and found a steady increase over time in implant-based, LDF and abdominal flap breast reconstruction, which was consistent with the trends observed in America (4, 11, 12). The scale of autologous breast reconstruction is affected by IBBR, and it has reached a relatively stable state. According to a nationwide cross-sectional survey of 110 hospitals in China, the proportion of breast reconstruction procedures performed after mastectomy was 10.7%, with 70% being implant-based reconstruction, 17% being autologous tissue reconstruction, and 13% being a combination (13). Consistent with changes in MD Anderson’s reconstruction methods over the past ten years, the number of free flaps for breast reconstruction has steadily increased as the use of prostheses has increased, which is an international trend (14). In Europe, Petit et al. showed that autologous breast reconstruction was used in approximately 20% of all reconstructions (15). This finding suggested that the advantages of autologous reconstruction has continued to be affirmed. In autologous reconstruction, the patient’s own tissue is used, thus ensuring a breast that appears and feels more natural, is permanent, better withstands the aging process and better tolerates radiation.

The proportion of LDFs used in autologous breast reconstruction patients also decreased each year and decreased to a relatively stable proportion of approximately 65.0% after 2013. The proportion of breast reconstruction using abdominal flaps has increased over time, and the trend is expected to surpass LDF thus becoming the most used autologous flap. However, the latissimus dorsi flap is still the most used autologous flap for breast reconstruction in China. This was in contrast to the situation in the United States, where abdominal flaps were most commonly used in autologous reconstruction (16). In Europe, LDF and TRAM flaps are the most frequently used for breast reconstruction (17). Compared with the data from MROC, patients undergoing LDF reconstruction in the study were younger than those in America (37 vs. 53.5 years old). The mean age of patients who underwent reconstruction using a LDF was younger than that of those receiving a DIEP, which is in contrast to the results of MROC (16). The proportion of delayed reconstruction and radiotherapy was lower than that in American patients (16). The LDF has a small amount of tissue and can be combined with the implant to reconstruct a satisfactory breast shape. For American patients with larger or more ptotic breasts desiring reconstruction, a single LDF may be insufficient in providing enough tissue volume, while DIEP might be more sufficient in providing adequate tissue volume (5, 18). Compared with breast reconstruction using an abdominal flap, LDF with/without implants breast reconstruction is advantageous in that the operation is simple and safe, produces a concealed back scar, and can fill subclavian defects and form breast axillary folds, which is especially suitable for patients who have not given birth and wish to have children (9). The comparative study performed by Lee et al. showed that the risk of complications of LDF breast reconstruction was similar to that of abdominal-based autologous breast reconstruction, despite having a shorter operative time than abdominal-based autologous breast reconstruction (19). In addition, the learning curve of surgeons for latissimus dorsi reconstruction is relatively short, which is more conducive to its adoption in local hospitals. The extensive use of prostheses has jointly improved the popularity of LDF in China.

LDF is a solid and reliable donor site for autologous breast reconstruction, and it has wide applicability in breast reconstruction. A LDF is used more often for immediate breast reconstruction and less often for delayed reconstruction. This may be related to the relatively small tissue volume of the LDF, which is insufficient for delayed reconstruction. The LDF can be easily transposed to the anterior chest for primary breast reconstruction and coverage of chest wall defects, for salvage of previous flap failure, such as abdominal flap necrosis or partial necrosis, and for salvage of implant reconstruction failure, exposed expander/implant or as part of a combined approach (20, 21). It can be used for oncoplastic surgery, especially in volume replacement technology.

There are two main technical modalities of LDF breast reconstruction, including ELDF and LDF with implant reconstruction. In our study, we found that age and BMI were independent factors influencing the reconstruction options, and older patients or those with a higher BMI tended to receive an ELDF. There was no significant difference in choosing radiotherapy or neoadjuvant chemotherapy. Considering that patients with more metastatic lymph nodes are more likely to receive subsequent radiotherapy, surgeons tend to choose extended LDF breast reconstruction rather than LDF combined with implants. A prospective study performed by Cowen et al. showed that T3 or T4 tumors (P = 0.0005), smoking habit (P = 0.001), and pN+ axillary status (P = 0.004) were significant factors associated with IBBR failure after radiotherapy (22). In this study, LDF reconstruction with implants did not increase the duration of hospitalization or decrease the rate of adjuvant radiotherapy. Leuzzi S et al. showed that there was no significant difference in the hospitalization duration of patients receiving a LD flap with an implant or lipofilling, which is consistent with this study. However, they found that the surgical complication rate was higher in patients undergoing LDF combined with implant reconstruction (14.2% vs. 18.8%), which was not observed in this study (major complications of LDF vs. LDF+implant: 2.6% vs. 1.9%, *P*=0.542).

Advantages of the LDF used for breast reconstruction include minimal donor site morbidity, relatively quick recovery, and reasonably good aesthetic outcome. A previous study showed that only 2.29% of patients experienced major complications, suggesting that LDF combined with or without implant breast reconstruction was reliable and safe. Berthet G et al. suggested that immediate breast reconstruction using LDF appeared to have excellent tolerance to subsequent radiotherapy, and adjuvant radiotherapy had no impact on patient aesthetic satisfaction (23). Even combined with prosthetic reconstruction, the latissimus dorsi muscle can provide better prosthesis coverage compared with the mesh, which greatly reduces the rate of postoperative infection and prosthesis exposure. Patients who underwent LDF with lipofilling had a higher BREAST-Q score (6). Santosa KB et al. performed a prospective, multicenter trial to determine the outcomes of patients undergoing IBBR or autologous breast reconstruction using Breast-Q. The study showed that patients who underwent autologous reconstruction were more satisfied with their body (*P*<0.001) and had better psychosocial well-being (*P*=0.002) and sexual well-being (*P*<0.001) at 2 years postoperatively (24). Several studies have shown that patients with LDF flaps or rectus abdominis flap breast reconstruction have similar satisfaction scores (25, 26).

In this cohort study with a large number of patients, we comprehensively and reliably described the current development status of latissimus dorsi breast reconstruction in China. However, there were still several limitations. First, the inherent bias caused by a single-center retrospective study is inevitable. Second, seroma is an important complication after a latissimus dorsi (LD) flap procedure, but due to the limitation of retrospective studies, we failed to obtain the incidence of seroma in these patients. Aesthetics outcomes are indeed one of the important evaluation criteria for breast reconstruction surgery, but the aesthetics evaluation is relatively subjective. The lack of patient-reported outcomes made it impossible to analyze the development status of latissimus dorsi reconstruction with or without implants from the patients’ perspective. We will perform relevant studies on the patient-reported outcomes of patients with breast reconstruction in the future and compare the aesthetics outcomes of patients who received ELDF with those who received LDF with implants.

## Conclusion

The latissimus dorsi flap with or without implant breast reconstruction is a well-developed and safe reconstruction procedure performed in our center. Whether combined with implant reconstruction, the duration of postoperative hospitalization nor the incidence of major complications was affected.

## Data availability statement

The raw data supporting the conclusions of this article will be made available by the authors, without undue reservation.

## Ethics statement

The studies involving human participants were reviewed and approved by the Ethics Committee Review Board of the Fudan University Shanghai Cancer Center (Shanghai, China; ID: 1612167-18). Written informed consent for participation was not required for this study in accordance with the national legislation and the institutional requirements.

## Author contributions

SZ: Conceptualization, investigation, writing–original draft and writing–review, and editing. SH: Help analysis data and draft–review. SZ and SH contribute equally to this article and were considered co-first authors. JC, BY, XH, GL, YZ: Help to collect the data. JW: Supervise the planning and design of the study; data collection; statistical analysis and data interpretation; have full access to all the data in the study and be responsible for the integrity of the data and the accuracy of the data analysis; and manuscript review, revision, and reporting. All authors contributed to the article and submitted and approved the submitted section.
